# Incidence of Prediabetes and Diabetes in a European Longitudinal General Population Cohort and Its Associated Factors—Results From the Austrian LEAD Study

**DOI:** 10.1155/jdr/5540276

**Published:** 2025-04-22

**Authors:** Amiria Dal Grande, Maarten Van Herck, Robab Breyer-Kohansal, Tobias Mraz, Ahmad Karimi, Mohammad Azizzadeh, Sylvia Hartl, Otto C. Burghuber, Emiel F. M. Wouters, Alexandra Kautzky-Willer, Caspar Schiffers, Marie K. Breyer

**Affiliations:** ^1^Department of Internal Medicine, Protestant Hospital, Vienna, Austria; ^2^Ludwig Boltzmann Institute for Lung Health, Vienna, Austria; ^3^NUTRIM, School of Nutrition and Translational Research in Metabolism, Maastricht University Medical Center, Maastricht, The Netherlands; ^4^Faculty of Medicine, Sigmund Freud Private University, Vienna, Austria; ^5^Department of Respiratory and Pulmonary Diseases, Clinic Hietzing, Vienna Healthcare Group, Vienna, Austria; ^6^Department of Respiratory and Pulmonary Diseases, Site Penzing of Clinic Ottakring, Vienna Healthcare Group, Vienna, Austria; ^7^Division of Endocrinology and Metabolism, Department of Internal Medicine III, Medical University of Vienna, Vienna, Austria

**Keywords:** cohort study, diabetes, glycaemic status, incidence, population-based study, prediabetes, trajectories, undiagnosed diabetes

## Abstract

**Aims:** This study evaluates the incidence of (pre)diabetes in an Austrian population over a broad age span and addresses whether alterations in lifestyle, blood markers, and body composition are associated with the development of (pre)diabetes.

**Material and Methods:** Data from the first and second phases of the Austrian LEAD study, a longitudinal population–based cohort study, were used. Inclusion criteria were a valid glycaemic status (i.e., normoglycaemia, prediabetes, and diabetes) at both phases using American Diabetes Association criteria. Besides blood samples, body composition parameters and an interviewer-administered questionnaire were assessed. A binary logistic regression was performed to answer the research question.

**Results:** In total, 7822 individuals (51% females, 46 ± 19 years with 9.6% aged < 18 years, median follow-up time 4.1 [3.9–4.5] years) were included. The overall incidence rate was estimated at 63.0 (95% CI [59.7; 66.3]) and 8.4 (95% CI [7.4; 9.5]) per 1000 person-years for prediabetes and diabetes, respectively. In the 6–<10-, 10–<20-, and 20–<30-year age bins, an incidence rate of, respectively, 30.2, 16.3, and 13.4 per 1000 person-years (prediabetes) and 2.0, 3.5, and 1.3 (diabetes) was observed. Further, 38.3% of diabetic individuals at Visit 2 were undiagnosed and thus untreated. Factors identified as being significantly associated with progression towards (pre)diabetes included changes in triglycerides, high-density lipoprotein cholesterol, total cholesterol, and visceral adipose tissue mass, besides male sex, older age, low education level, and urban residence.

**Conclusions:** The overall incidence of (pre)diabetes in the Austrian population is high and highlights the need for screening from a young age and on a regular basis so that preventive and treatment strategies can be implemented at an early stage.

**Trial Registration:** ClinicalTrials.gov identifier: NCT01727518

## 1. Introduction

Diabetes is associated with increased morbidity and mortality rates [1, 2]. The illness is a fast-growing global health concern with tremendous healthcare costs and significant negative socioeconomic impact [3]. Diabetes ranks among the leading causes of disability-adjusted life years (DALYs) worldwide in adults aged 50–75 and older [1], and a further increase in the global diabetic burden is forecasted [4].

Approximately 10.5% of the global population aged 20–79 years lived with diabetes worldwide in 2021, and this is expected to rise to 12.2% by 2045 [4]. Additionally, 6.2%–10.6% of adults worldwide have prediabetes [5], defined as a higher-than-normal blood glucose level but below the level of diagnostic criteria for diabetes [6]. This is of particular concern as it is highly prevalent, poses a risk of developing Type 2 diabetes, and is related to diabetes-related comorbidities and a higher all-cause mortality [7, 8]. Nevertheless, it remains mostly asymptomatic and thereby unnoticed. As prediabetes is not a fixed state [7, 9], it is a useful target to implement preventive strategies. Although the prevalence of (pre)diabetes is known to increase with age [5], it also occurs in adolescents and young adults [5, 10]. Previously, an unexpected 4%–7% prevalence rate of prediabetes in children and adolescents was found [11]. Moreover, youth-onset diabetes is associated with early diabetes-related complications [12] and excess mortality compared to adult-onset diabetes [13].

Given the huge impact on individual and public health, country-specific and representative data for the incidence of prediabetes and diabetes over a broad age span are of importance to target interventions and preventive strategies. However, epidemiologic data from studies in the general population are scarce [5], and screening is often started at a higher age [6]. Furthermore, studies are often based on administrative sources and registries, thereby not accounting for undiagnosed (pre)diabetes. Additionally, only a few studies have a longitudinal design and can report on incidence and disease dynamics [5, 14].

Further, several factors (e.g., lifestyle, blood markers, and body composition) have been associated with (pre)diabetes in cross-sectional studies, but longitudinal studies that assessed these relationships are scarce [15].

Therefore, the primary objective of this study is to evaluate the incidence of prediabetes and diabetes in an Austrian longitudinal population–based cohort aged 6–>80 years. As a secondary objective, we aim to address whether alterations in lifestyle, inflammation, lipid profiles, and body composition are associated with the development of prediabetes or diabetes.

## 2. Methods

### 2.1. Study Design and Population

The Austrian LEAD (Lung, hEart, sociAl, boDy) study is an ongoing, unicentric, observational, longitudinal (visits every 4 years), population-based cohort study including a random sample of the Austrian population aged 6–>80 years. The study population consists of stratified samples from Vienna (urban cohort) and Lower Austria (rural cohort). Details about the study have been described elsewhere [16].

Longitudinal data from participants aged 6–>80 years taking part in the first (2012–2016) and second phase (2017–2021) of the study were used. Inclusion criteria for the current analysis were a valid glycaemic status at both visits, which is based on blood measurements consisting of glycated haemoglobin (HbA1c), fasting plasma glucose (FPG), and/or information on the intake of glucose-lowering medication. The LEAD study was approved by the local ethics committee of Vienna (EK-11-117-0711). Written informed consent was obtained from all participants. For participants under the age of 18, written consent was signed by their parents or legal representatives.

### 2.2. Data Collection

Both study visits took place at the LEAD study centre following a strict protocol. The study protocol required participants to have fasted for at least 8 hours. A team of trained study nurses ensured correct data collection. Both visits included the collection of venous blood samples, physical measurements including anthropometric measurements, and interviewer-based questionnaires.

### 2.3. Variables

#### 2.3.1. Glycaemic Status

Venous blood samples were analysed for FPG (in milligrams per deciliter) and HbA1c (in percent) to assess glycaemic status. Intake of glucose-lowering medication was assessed by questionnaire and included all types of insulin, insulin sensitizers, incretin mimetics, dipeptidyl peptidase inhibitors, glinides, sulfonylureas, biguanide derivatives, sodium-glucose transport protein 2 inhibitors, and alpha-glucosidase inhibitors.

Prediabetes and diabetes were defined according to current American Diabetes Association (ADA) definitions [6]. The diagnostic criteria used to define normoglycaemia (NBG), prediabetes, and diabetes are shown in Supporting Information 1: Table S1. Individuals with diabetes were divided into diagnosed (i.e., having a doctor's diagnosis or taking glucose-lowering medication) or undiagnosed diabetes (i.e., based on blood glucose measures and in the absence of glucose-lowering medication and a doctor's diagnosis). For the latter, the question “Do you have diabetes?” and the follow-up question “Has the disease [diabetes] been diagnosed by a doctor?” was used.

#### 2.3.2. Anthropometrics

A dual-energy x-ray absorptiometry (DXA) scan (Lunar Prodigy, GE Healthcare software enCORE) was performed to assess body composition (fat mass (FM), lean mass (LM), appendicular lean mass (ALM), and visceral adipose tissue (VAT)). Further, body weight, height, and waist circumference were measured. Body mass index (BMI; in kilograms per square meter) was calculated for adults and as fitted body mass index (fBMI; in kilograms per cubic meter) for participants < 18 years [17]. Obesity was classified according to the WHO definition as a BMI ≥ 30 kg/m^2^ (adults) and as two standard deviations (SDs) above the mean BMI-for-age value (< 18 years) [18].

#### 2.3.3. Other Measures

Demographics such as age and sex were assessed. Age was used both as a continuous and categorical variable (i.e., stratified in 10-year age bins starting from 6–<10 to 70+ age bins).

Besides blood glucose levels (i.e., FPG and HbA1c), blood lipids (low-density lipoprotein cholesterol (LDL-C), high-density lipoprotein cholesterol (HDL-C), and triglycerides) and inflammation markers (high-sensitivity C-reactive protein (hsCRP) and fibrinogen) were also assessed.

Lifestyle habits were assessed by interviewer-based questionnaires, including information on nutrition (healthy/unhealthy), physical activity (PA) in minutes per day, smoking status (never/former/current smoker), smoking history in pack-years, regular alcohol consumption (yes/no), place of residence (urban/rural), household income (low/intermediate to high), and education level (low/intermediate to high).

See Supporting Information 2: Data S1 for a detailed description of the assessments, questionnaires, (categorisation of) variables, and cut-off scores used to define datapoints as clinically nonfeasible. Of note, clinically nonfeasible datapoints were handled as missing data for that variable.

### 2.4. Statistics

#### 2.4.1. Incidence Calculation

To calculate the incidence of (pre)diabetes and any dysglycaemia (i.e., prediabetes or diabetes) between Visits 1 and 2, the population at risk for the condition was defined based on their glycaemic status at Visit 1. Consequently, incidence rates were calculated from (1) NBG to prediabetes, (2) NBG to any dysglycaemia, and (3) NBG or prediabetes to diabetes and were expressed per 1000 person-years of follow-up time. The number of events in each group at risk was divided by the follow-up time (i.e., the sum of person-years at risk) and multiplied by 1000. Person-years at risk per individual timeframe were calculated as full time of not having the condition (i.e., time between Visits 1 and 2; in case no event occurred between Visits 1 and 2) and half of the time in between visits (in case the event occurred between Visits 1 and 2). Regarding the incidence calculation of prediabetes, individuals who developed diabetes between Visits 1 and 2 were considered as a “no event” (i.e., they do not fulfil the ADA criteria for prediabetes as mentioned in Supporting Information 1); by that, they contributed to the person-years in the denominator but not to the number of events in the numerator. Further, we calculated the incidence rate for any dysglycaemia, which captures the occurrence of “prediabetes” or “diabetes” as an event. See Supporting Information 2 for further information on the calculation of the incidence rate.

#### 2.4.2. Trajectories in Glycaemic Status

The nine possible trajectories in glycaemic status between Visits 1 and 2 and their group size relative to their original group at Visit 1 are calculated. The nine possible trajectories of glycaemic status between Visits 1 and 2 are (1) NBG to NBG (i.e., normoglycaemic stable individuals), (2) NBG to prediabetes, (3) NBG to diabetes, (4) prediabetes to NBG, (5) prediabetes to prediabetes, (6) prediabetes to diabetes, (7) diabetes to NBG, (8) diabetes to prediabetes, and (9) diabetes to diabetes. Of note, Groups 2, 3, and 6 are aggregated in certain analyses as individuals that progressed towards prediabetes or diabetes (i.e., showing a deterioration of their glucose metabolism).

#### 2.4.3. Descriptives

Continuous variables were reported as mean with SD or median with interquartile range (IQR), and categorical variables as count with percentages (percent). Baseline characteristics were reported for the total group and for males and females separately. Total group and sex- and age-specific (in 10-year groups) incidence rates with corresponding 95% confidence intervals (95% CIs) using normal approximation were calculated. A priori, the level of significance was 0.05. Statistical analyses were performed using SPSS v28.0 and R Version 4.2.2. Visualisations were made using GraphPad Prism 9.3.1.

Binary logistic regression analysis was performed to identify changes in variables associated with developing a worse glucose metabolism between Visits 1 and 2 (i.e., NBG to prediabetes/diabetes or prediabetes to diabetes); staying normoglycaemic was set as the reference group. Odds ratios (ORs) and their 95% CIs were used to quantify the associations. For covariates included in the model, the change between visits (delta parameters, i.e., Visit 2 minus Visit 1) was calculated. The model was adjusted for sex, age, education, household income, place of residency, and nutrition at Visit 1. In addition, a second model without participants on statins at Visits 1 and/or 2 was performed to explore the influence of statins on blood lipids, as statins lower LDL-C and increase HDL-C [19, 20]. Of note, the regression analysis was performed for adults only. A combined analysis (with both individuals < 18 years and adults) and a separate analysis (with individuals < 18 years only) were not possible due to the difference in variables (i.e., body composition–related variables) and few observations in this cohort, respectively.

## 3. Results

Of the 11,423 participants, 10,930 individuals had a valid glycaemic status at Visit 1. From these, 7822 individuals were present in the current study (Supporting Information 3). Reasons for noninclusion in the present study were loss to follow-up (*n* = 2921) or invalid glycaemic status at Visit 2 (*n* = 187). The characteristics of these participants (*n* = 3108) can be found in Supporting Information 4. In general, excluded individuals were, on average, slightly younger and more often had a low education level and low household income than included individuals.

### 3.1. Cohort Characteristics

The study population had a mean age of 46 ± 19 years, with 751 participants aged 6–<18 years. Slightly more females than males were represented in the total sample (51% vs. 49%, respectively), which was the opposite in participants aged 6–<18 years (48% vs. 52%, respectively). The median follow-up time was 4.1 years [3.9–4.5] (range 3.0–9.5 years). Males showed higher mean blood glucose values (HbA1c and FPG), waist circumference, prevalence of obesity, and mean BMI (adults) but not fBMI (< 18 years) compared to females (Table 1).

### 3.2. Glycaemic Status and Different Trajectories

**Figure 1:**
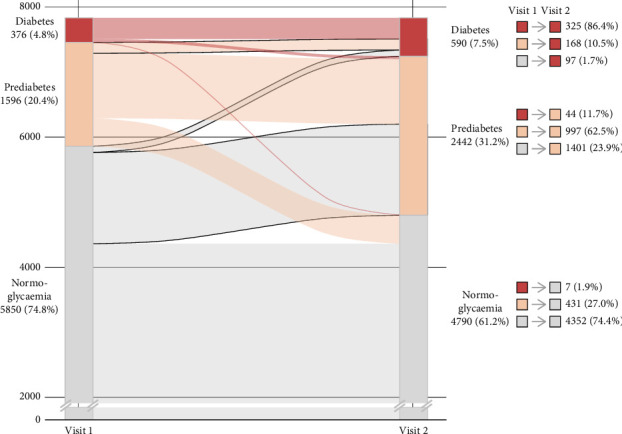
Alluvial plot showing glycaemic status at Visits 1 and 2 according to the current American Diabetes Association criteria. The width of the lines in the figure is proportional to the flow rate. For each trajectory, the absolute number of individuals and corresponding percentage as part of their glycaemic status at Visit 1 is reported.

Following a median time of 4.1 years, the overall prevalence of prediabetes and diabetes in this cohort increased from Visit 1 (20.4%, 95% CI [19.5; 21.3], and 4.8%, 95% CI [4.3; 5.3], respectively) to Visit 2 (31.2%, 95% CI [30.2; 32.2], and 7.5%, 95% CI [7.0; 8.1], respectively). The share of undiagnosed individuals with diabetes increased from 25.3% at Visit 1 to 38.3% at Visit 2. From the individuals with undiagnosed diabetes at Visit 1, 37.2% went into remission, whereas 28.7% remained undiagnosed and untreated at Visit 2 (i.e., no formal doctor's diagnosis and no diabetes medication), and 34.0% of the individuals with undiagnosed diabetes at Visit 1 were diagnosed at Visit 2. Further, the alluvial plot with trajectories of glycaemic status between Visits 1 and 2 revealed that 23.9% and 1.7% of the individuals with NBG progressed to prediabetes and diabetes, respectively, while 10.9% of the individuals with prediabetes progressed to diabetes. In addition, only a minority of the individuals with diabetes at Visit 1 regressed to prediabetes or NBG (11.7% and 1.9%, respectively), whereas 27.0% of the individuals with prediabetes regressed to a normoglycaemic state (Figure 1).

### 3.3. Incidence Trends of Prediabetes, Diabetes, and Any Dysglycaemia

**Figure 2:**
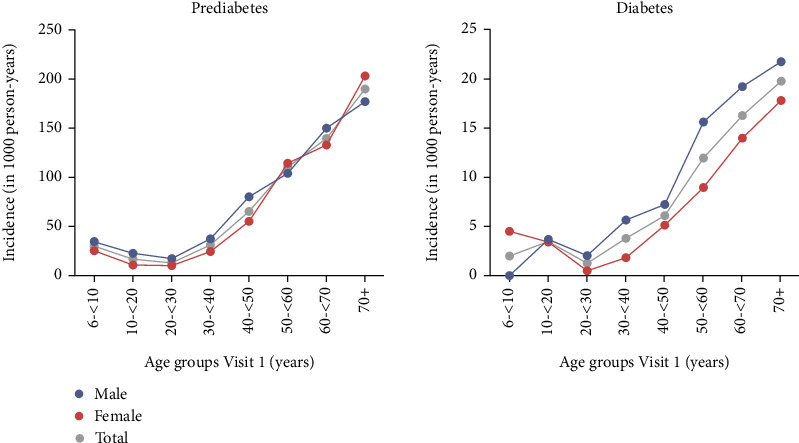
Incidence of diabetes and prediabetes per 1000 person-years stratified by age and sex. Blue lines represent males, and red lines represent females. Age from 6 to <10 years and in decades from 10 to 70+ years (*x*-axis).

The overall incidence rate for prediabetes was 63.0 (95% CI [59.7; 66.3]) per 1000 person-years and ranged from 30.2 (95% CI [18.8; 41.6]) in the 6–<10-year age group up to 189.9 (95% CI [162.0; 217.8]) in the 70+ age group. The lowest incidence rate was observed in the 20–<30-year age group (13.4, 95% CI [9.7; 17.2]; Figure 2; Supporting Information 5).

The incidence of diabetes followed a similar pattern, although at a lower rate. The overall incidence rate for diabetes was 8.4 (95% CI [7.4; 9.5]) per 1000 person-years and ranged from 2.0 (95% CI [0.0; 4.8]) in the 6–<10-year age group up to 19.8 (95% CI [14.2; 25.3]) in the 70+ age group. The lowest incidence rate was observed in the 20–<30-year age group (1.3, 95% CI [0.2; 2.3]; Figure 2; Supporting Information 5). The incidence of prediabetes, diabetes, and any dysglycaemia stratified for age and sex follows the same pattern as described above and can be found in Supporting Information 5 and in more detail in Supporting Information 6, 7, and 8, respectively. The incidence for any dysglycaemia was overall 68.0 (95% CI [64.6; 71.5]) per 1000 person-years and ranged from 32.6 (95% CI [20.7; 44.5]) in the 6–<10-year age group up to 205.7 (95% CI [176.3; 235.1]) in the 70+ age group (Supporting Information 5).

### 3.4. Associated Factors of Progressing Towards Prediabetes or Diabetes in Adults

Binary logistic regression was used to analyse the relationship between alternations in lifestyle, inflammation, lipid profile, and anthropometry and the development of (pre)diabetes. BMI was excluded from the analysis because of strong collinearity with the anthropometric parameters of the DXA scan. Factors that were identified as being significantly associated with progressing towards prediabetes or diabetes were change in triglycerides, change in HDL-C, change in cholesterol, and change in *z*-score of VAT mass, and this was independent of the levels of inflammation markers hsCRP and fibrinogen, and corrected for confounding variables age, sex, education level, place of residence, household income, and nutrition at Visit 1 (Table 2). When holding all other predictor variables constant, the odds of progressing towards (pre)diabetes increased by 42.0% (OR 1.420, 95% CI [1.165; 1.729]) for each *z*-score increase in change of VAT mass, by 0.3% (OR 1.003, 95% CI [1.001; 1.004]) for each milligram per deciliter increase in change of triglycerides, and by 1% (OR 1.010, 95% CI [1.002; 1.018]) for each milligram per deciliter increase in change in HDL-C. Further, the odds of progressing towards (pre)diabetes decreased by 0.5% (OR 0.995, 95% CI [0.993; 0.998]) for each milligram per deciliter increase in change of cholesterol. The results of the logistic regression model without individuals on statins at Visit 1 and/or Visit 2 were similar (Supporting Information 9).

## 4. Discussion

The LEAD study is the first population-based study examining the incidence of prediabetes and diabetes in a large cohort that includes both children and adolescents, as well as an adult population. The prevalence of both prediabetes and diabetes increased over a 4-year follow-up period among the participants with a valid glycaemic status to 31.2% and 7.5%, respectively. The overall incidence of prediabetes and diabetes was high: 63.0 and 8.4 per 1000 person-years, respectively. The development of an abnormal glycaemic status was more likely with an increase in VAT mass and not with an increase in the FM index, independent of levels of inflammation markers hsCRP and fibrinogen.

To date, only a few longitudinal studies have investigated the incidence rate of diabetes and prediabetes. Nevertheless, comparison is difficult due to the differences in study design, diagnostic criteria, and age range. Furthermore, most cohorts are limited to adults, although (pre)diabetes can be present at a young age [10]. In our cohort, the overall incidence rate for diagnosed and undiagnosed diabetes was 8.4 per 1000 person-years. The German public health institute found a comparable incidence rate for diagnosed and undiagnosed diabetes of 7.9 per 1000 person-years over a 12-year period, although the incidence estimation was done in adults only [21]. In addition, a retrospective cohort study in the UK found a lower incidence rate for diabetes (4.5 and 3.9 per 1000 person-years in males and females, respectively). Nevertheless, only Type 2 diabetes was recorded using routinely recorded primary care data, which could explain the lower incidence rate. Then again, a higher incidence rate per increase in age bin, along with a sex difference, was observed (i.e., a higher incidence rate for diabetes in males than females), which was also found in our cohort [22]. More recent country-specific incidence data for diabetes was published by the Austrian Ministry of Health. An overall incidence of 5.4 per 1000 person-years was found; however, undiagnosed individuals with diabetes were not considered, as prescription data of antidiabetic drugs was used [23].

In addition, the incidence of prediabetes was estimated in the current study. The overall incidence rate for prediabetes was 63.0 per 1000 person-years. In Asian–Indian adults, an incidence rate of 29.5 per 1000 person-years for prediabetes was found over a period of 9 years, although the cohort dates back from 2001 to 2013 [24], which could explain the discrepancy with our findings. More recently, an incidence rate of 18.1 per 1000 person-years was reported in Indian children and adolescents over a 7-year period [25], which is comparable to the prediabetes incidence observed in the 6–10- and 10–<20-year age groups in our cohort (30.2 and 16.3 per 1000 person-years, respectively). Nevertheless, it should be mentioned that comparison with data from other continents is not straightforward as racial/ethnic disparities are present in the development of (pre)diabetes [26].

The high rates of diabetes and prediabetes, even in children, adolescents, and young adults, give cause for concern. We hypothesize that transient insulin resistance in puberty leads to this surge in prediabetes in these age groups (and consequently in a few susceptible individuals also to diabetes). Insulin resistance peaks around the age of 12–13 years [27, 28]. After puberty, this state is reverted in many individuals [27]. Further, the conversion rate from prediabetes in Visit 1 to NBG in Visit 2 is 75% (*N* = 27) in children, whereas it is only 27% (*N* = 431) in the adult cohort (data not shown).

To date, screening for abnormal glucose levels and diabetes is recommended by the US Preventive Services Task Force (USPSTF) in asymptomatic individuals from the age of 35 if overweight or obesity is present [29, 30]. Even though children, adolescents, and young adults can benefit from early detection, prevention, and treatment of (pre)diabetes. In our cohort, an incidence rate for prediabetes of 30, 16, 13, and 31 per 1000 person-years was found in the 6–<10-, 10–<20-, 20–<30-, and 30–<40-year age bins, respectively. These numbers provide a clear rationale for screening and implementation of treatment strategies at an early age. In addition, 38% of patients that fulfilled the ADA criteria for diabetes at Visit 2 were undiagnosed cases (compared to 25% at Visit 1) with the highest proportion of undiagnosed cases in the 6–<10- to 40–<50-year age bins (data not shown). These findings highlight the need for proper screening across all ages and on a regular basis, as drug-free remission of diabetes is more likely to be achieved in individuals with a shorter disease duration [31, 32].

The logistic regression model identified changes in triglycerides, HDL-C, total cholesterol, and VAT mass as being significantly associated with progressing towards (pre)diabetes, whereas changes in FMI as well as changes in inflammation markers hsCRP and fibrinogen were not associated with progressing towards (pre)diabetes. The relationship between an increase in VAT [33, 34] and triglycerides [35], on the one hand, and developing an impaired glycaemic status on the other hand, is established in the literature. Further, the confounders (higher age), sex (male), education level (low), and place of residence (rural) were identified as significant predictors of the development of an impaired glycaemic status, as reported in the literature [36]. Current findings highlight the importance of preventive measures like regular PA, a healthy diet to prevent weight gain, and self-monitoring of food intake and weight. The findings regarding the increase in HDL-C over time and increased likelihood to develop an impaired glycaemic status, and the increase in total cholesterol and decreased likelihood to develop an impaired glycaemic status, are less straightforward. These alterations can be a consequence of the metabolic state, but another possible explanation is the use of certain medications like statins that lower LDL-C and increase HDL-C [19, 20]. However, the logistic regression model with and without individuals on statins at Visit 1 and/or Visit 2 is comparable, which indicates that statins did not mediate the results. Our findings are consistent with data from other epidemiologic and genetic studies showing that lower levels of LDL-C are associated with a higher risk of diabetes [37, 38]. Furthermore, the significant association of delta VAT but not FMI illustrates the importance of monitoring VAT, a unique hormonally active component of total body fat, to a greater extent than FM in the metabolic workup. Also, the nonsignificant relationship between the change in inflammation markers hsCRP and fibrinogen and the likelihood of progressing towards (pre)diabetes is of interest. Recent evidence suggests low-grade inflammation as a potential determinant of the development of diabetes [39]. However, the present analysis challenges this notion by suggesting that metabolic changes may be the underlying factors contributing to the development of (pre)diabetes.

### 4.1. Strengths and Limitations

The current unicentric study used longitudinal data from around 8000 individuals to estimate the incidence of diabetes and prediabetes. A country-specific and representative sample of the Austrian population was used for the current study, which was not limited to adults only but also included children and adolescents. Nevertheless, there is evidence that the incidence of diabetes can vary in Austrian regions [40]. Further, glucose markers from venous blood sampling and prospectively collected information about medical history and medication intake were used to define individuals' glycaemic status, allowing estimation of the true incidence rate that considers both diagnosed and undiagnosed cases. The glycaemic status was determined using the ADA criteria. Despite the fact there is no real consensus on the diagnostic criteria of prediabetes, these criteria are widely accepted [41]. Although it must be said that the ADA criteria use a lower cut-off value to define prediabetes via FPG and HbA1c, which results in a higher prevalence and consequently a higher incidence rate [41]. Further, excluded individuals more often had a low education level and low household income compared to included individuals. As both factors are related to the development of diabetes, this could have influenced our results to a certain extent [36]. Our results suggest that a change in PA does not affect glycaemic state; further studies considering metabolic equivalent of tasks (METs) will be needed to delineate the precise role of PA. Over a 4-year follow-up, the prevalence of undiagnosed (and thereby untreated) diabetes increased from 25% to 38%. This is despite the fact individuals identified with undiagnosed diabetes at Visit 1 were informed about their abnormal glycaemic status and advised to see a general practitioner. Therefore, it is likely that the observed percentage of undiagnosed diabetes at Visit 2 is an underestimation. Lastly, it should be mentioned that a part of Phase 2 data was gathered after March 2020 (i.e., the start of the COVID-19 pandemic). Emerging evidence suggests that COVID-19 may lead to new-onset diabetes, although more research is needed to confirm this [42, 43]. In our cohort, such a trend could not be detected (data not shown).

## 5. Conclusion

The incidence rate for prediabetes and diabetes in the general Austrian population over a 4-year follow-up period is 63.0 and 8.4 per 1000 person-years. High incidence rates were observed in all age bins, including in children, adolescents, and young adults. A large share of the population with diabetes was undiagnosed, which highlights the importance of regular screening at a broad age span, starting with the very young ones. Furthermore, the significant association of delta VAT but not FMI illustrates the importance of monitoring VAT to a greater extent than FM in the metabolic work-up.

## Figures and Tables

**Table 1:** Baseline characteristics of the total study population and stratified by sex and age.

	**Total**						**< 18 years**						**+18 years**					
	**N**	**All**	**n**	**M**	**n**	**F**	**N**	**All**	**n**	**M**	**n**	**F**	**N**	**All**	**n**	**M**	**n**	**F**
Age (years)	7822	45.7 (18.5)	3806	45.4 (18.7)	4016	46.0 (18.2)	751	12.4 (3.3)	390	12.3 (3.5)	361	12.5 (3.2)	7071	49.2 (15.7)	3416	49.2 (15.8)	3655	49.3 (15.5)
HbA1C (%)	7807	5.3 (0.5)	3795	5.3 (0.5)	4012	5.2 (0.5)	751	5.0 (0.3)	390	5.1 (0.3)	361	5.0 (0.3)	7056	5.3 (0.5)	3405	5.3 (0.6)	3651	5.2 (0.5)
Fasting plasma glucose (mg/dL)	7813	93.1 (15.5)	3800	95.6 (17.1)	4013	90.7 (13.3)	751	86.1 (8.0)	390	86.9 (7.7)	361	85.3 (8.2)	7062	93.8 (15.9)	3410	96.6 (17.6)	3652	91.2 (13.5)
BMI in adults (kg/m^2^)	NA	NA	NA	NA	NA	NA	NA	NA	NA	NA	NA	NA	7071	25.9 (4.7)	3416	26.7 (4.2)	3655	25.2 (5.1)
Fitted BMI in participants < 18 years (kg/m^3^)	NA	NA	NA	NA	NA	NA	751	12.5 (2.0)	390	12.4 (1.9)	361	12.6 (2.0)	NA	NA	NA	NA	NA	NA
Waist circumference (cm)	7811	91.7 (14.9)	3802	95.9 (14.7)	4009	87.7 (14.0)	751	70.6 (11.6)	390	71.1 (12.2)	361	70.0 (10.9)	7067	93.8 (13.7)	3414	96.7 (12.3)	3653	89.3 (13.5)
Obesity^a^, *n* (%)	7812	1229 (15.7)	3803	665 (17.5)	4009	564 (14.1)	740	11 (1.5)	387	6 (1.6)	354	5 (1.4)	7071	1218 (17.2)	3416	659 (19.3)	3655	559 (15.3)

*Note:* Data is reported as mean and standard deviation (for age, HbA1c, fasted plasma glucose, BMI in adults, fitted BMI in participants < 18 years, and waist circumference) or number and percentage (for obesity).

Abbreviations: BMI, body mass index; HbA1c, glycated haemoglobin; NA, not applicable.

^a^Presence of obesity is based on a BMI = 30 kg/m^2^ (adults) and as > 2 standard deviations (SDs) above the mean BMI-for-age value mean (< 18 years).

**Table 2:** Multivariate binary logistic regression analysis of the association between physical activity, serum markers, and anthropometrics and individuals developing a worse glycaemic status referent to individuals staying normoglycaemic in an adult population.

**Variables**	**B** ** estimate**	**SE**	**z** ** -statistic**	**OR [95% CI]**	**p** ** value**
(Intercept)	−4.217	0.196	−21.552	0.015 [0.010; 0.022]	**< 0.001**
Delta physical activity (min/day)	−0.000	0.000	−0.660	1.000 [0.999; 1.000]	0.509
Delta smoking (pack years)	0.001	0.005	0.312	1.001 [0.992; 1.011]	0.755
Delta triglycerides (mg/dL)	0.003	0.001	3.891	1.003 [1.001; 1.004]	**< 0.001**
Delta HDL-C (mg/dL)	0.010	0.004	2.458	1.010 [1.002; 1.018]	**0.014**
Delta hsCRP (mg/dL)	0.015	0.010	1.417	1.015 [0.995; 1.036]	0.156
Delta fibrinogen (g/L)	0.012	0.077	0.151	1.012 [0.871; 1.175]	0.880
Delta cholesterol (mg/dL)	−0.005	0.001	−3.437	0.995 [0.993; 0.998]	**0.001**
Delta FMI (*z*-score)	0.018	0.132	0.135	1.018 [0.768; 1.319]	0.892
Delta LMI (*z*-score)	0.123	0.138	0.885	1.130 [0.862; 1.483]	0.376
Delta ALMI (*z*-score)	−0.035	0.103	−0.337	0.966 [0.790; 1.183]	0.736
Delta VAT mass (*z*-score)	0.351	0.101	3.478	1.420 [1.165; 1.729]	**< 0.001**

Sex (female)	−0.227	0.083	−2.736	0.797 [0.677; 0.938]	**0.006**
Age at Visit 1 (years)	0.066	0.003	20.571	1.069 [1.062; 1.075]	**< 0.001**
Household income at Visit 1 (low)	0.130	0.137	0.954	1.139 [0.869; 1.486]	0.340
Education level at Visit 1 (low)	0.228	0.092	2.483	1.256 [1.049; 1.502]	**0.013**
Place of residence at Visit 1 (rural)	−0.262	0.106	−2.479	0.770 [0.625; 0.945]	**0.013**
Nutrition at Visit 1 (unhealthy)	0.168	0.094	1.791	1.183 [0.985; 1.422]	0.073

*Note:*Delta is calculated by subtracting Visit 1 from Visit 2 (i.e., Visit 2 minus Visit 1). The model was adjusted for the following confounders: sex, age at Visit 1, household income at Visit 1, education level at Visit 1, place of residence at Visit 1, and nutrition at Visit 1. *p* values in bold are significant (i.e., *p* value < 0.05).

Abbreviations: 95% CI, 95% confidence interval; ALMI, appendicular lean mass index; FMI, fat mass index; HDL-C, high-density lipoprotein cholesterol; hsCRP, high-sensitivity C-reactive protein; LMI, lean mass index; OR, odds ratio; VAT, visceral adipose tissue.

## Data Availability

The datasets generated and/or analysed during the current study are not publicly available as the population-based LEAD study is still ongoing, and output related to this article is expected in the future. However, data is available from the corresponding author upon reasonable request.

## Supporting Information

Supporting Information 1 Table S1: Diagnostic criteria from the American Diabetes Association to define glycaemic status.

Supporting Information 2 Data S1: Extra information on the methodology of the study.

Supporting Information 3 Figure S1: Flowchart of patient selection.

Supporting Information 4 Table S2: Characteristics of included and excluded individuals at Visit 1.

Supporting Information 5 Table S3: Incidence of prediabetes, any dysglycaemia, and diabetes per 1000 person-years in 10-year groups.

Supporting Information 6 Table S4: Incidence of prediabetes per 1000 person-years in 10-year groups stratified by sex.

Supporting Information 7 Table S5: Incidence of diabetes per 1000 person-years in 10-year groups stratified by sex.

Supporting Information 8 Table S6: Incidence of any dysglycaemia (prediabetes or diabetes) per 1000 person-years in 10-year groups stratified for sex.

Supporting Information 9 Table S7: Multivariate binary logistic regression analysis of the association between physical activity, serum markers, and anthropometrics in individuals developing a worse glycaemic status referent to individuals staying normoglycaemic in an adult population. Participants using statins in Visit 1 and/or 2 were excluded.
