# Supplementary material for: Incidence of Prediabetes and Diabetes in a European Longitudinal General Population Cohort and Its Associated Factors—Results From the Austrian LEAD Study
Source: J Diabetes Res. 2025 Apr 22;2025:5540276. doi: 10.1155/jdr/5540276 (PMC12041627; doi:10.1155/jdr/5540276)
Supplement: Supporting Information 1 — Table S1: Diagnostic criteria from the American Diabetes Association to define glycaemic status. [file 5540276.f1.docx]

**Supplemental material – Online supplement 1**

**Online Table 1.** Table with diagnostic criteria from the American Diabetes Association to define glycaemic status.

| **ADA criteria** | **FPG** |  | **HbA1c** |  | **Glucose-lowering medication *** |
| --- | --- | --- | --- | --- | --- |
| **Normoglycaemia** | <100 mg/dl  (i.e., <5.6mmol/L) | AND | <5.7%  (i.e., <39 mmol/mol) | AND | No intake |
| **Prediabetes** | 100-125mg/dl  (i.e., 5.6 - 6.9 mmol/L) | AND / OR | 5.7-<6.5%  (i.e., 39-<48 mmol/mol) | AND | No intake |
| **Diabetes** | ≥126 mg/dl  (i.e., ≥7.0 mmol/L) | AND / OR | ≥6.5%  (i.e., ≥48 mmol/mol) | AND / OR | Intake |
| * Glucose-lowering medication are defined as all types of insulin, insulin sensitizers, incretin mimetics (GLP-1 agonist), dipeptidyl peptidase (DPP4) inhibitors, glinides, sulfonylureas, biguanide derivates, sodium-glucose transport protein 2 (SGLT2) Inhibitors, and alpha-glucosidase inhibitors. Abbreviations: ADA, American Diabetes Association; FPG, Fasting Blood Glucose; HbA1c, glycated haemoglobin. | | | | | |
