# Supplementary material for: Incidence of Prediabetes and Diabetes in a European Longitudinal General Population Cohort and Its Associated Factors—Results From the Austrian LEAD Study
Source: J Diabetes Res. 2025 Apr 22;2025:5540276. doi: 10.1155/jdr/5540276 (PMC12041627; doi:10.1155/jdr/5540276)
Supplement: Supporting Information 2 — Data S1: Extra information on the methodology of the study. [file 5540276.f2.docx]

**Supplemental material - Online supplement 2**

**Online Methods 1.** Extra information on the methodology of the study.

**Research design and methods – data collection**

**Venous blood sampling**

Venous blood samples were analysed using the Sysmex XN1000 hematology analyzer, this was done by specialists at the Institute for Laboratory Medicine at Klinik Penzing, Vienna, Austria.

**Anthropometrics**

Body weight was measured by a high precision scale (exacta CLASSIC by SOEHNLETM).

Waist circumference was reported in cm for adults and individuals <18 years, clinical non-feasible outliers (i.e., waist circumference <25 cm) were identified and handle as missing data.

Standardized Dual-energy X-ray absorptiometry (DXA) scan (Lunar Prodigy, GE Healthcare®) was performed to assess body composition and analysed by enCORETM (Version 17, 2016). Body compartments were indexed to total body height and expressed as z-scores. The LEAD study reference values for adults were used. For participants <18 years, fitted Body compartment z-scores, with height indexed to different exponents (as compared to height^2^ for adults), were used. As VAT is not standardised for individuals <18 years, it could not be analysed for participants <18 years. No extreme outliers (i.e., >10 SD above/below the mean) were identified.

**Questionnaire data**

***Daily physical activity***

Daily physical activity (PA) was calculated by summing the time doing vigorous or moderate sports, physical work or locomotion by bicycle or foot, and expressed in minutes per day. The maximum of physical activity per day was set to 720 min/day, this included 99.5% of all observations within the cohort. Extreme outliers (n=37) were identified and handle as missing data.

***Smoking status and history***

Participants were classified as current, former, or never smoker. Smoking history is expressed in pack years in which one pack year equals the daily consumption of one pack of cigarettes (i.e., 20 pieces) for one year or 2 packs per day for half a year, and so on. It is calculated by multiplying the number of packs of cigarettes smoked per day by the number of years the person has smoked.

***Alcohol consumption***

Alcohol consumption was categorized into regular and irregular alcohol consumption, in which regular alcohol consumption is defined as the consumption of ≥1 glass of alcohol per day.

***Place of residence***

The place of residency at visit 1 was categorized into urban and rural.

***Education level***

Education level was categorized into ‘low’ and ‘intermediate to high’ education level. Individuals were classified as ‘intermediate to high’ level of education if upper secondary or tertiary education was completed. For participants <18 years, the education level of their parents was used.

***Household income***

Household income was categorized into ‘low’ and ‘intermediate to high’ household income, in which ‘low’ household income was defined as a monthly household income of <1100 euros. Household income is represented as equivalised monthly income, which is divided by the sum of person quantifiers in a household (first adult quantifier 1, further adults quantifier for each additional adult 0.5, each child quantifier 0.3). This method considers varying sizes and structures of households and is in line with the EU-Scala (Statistic Austria, Household income). For participants <18 years, information about monthly household years income information of their parents was used.

***Nutrition***

Nutrition was categorized into healthy and unhealthy, in which unhealthy nutrition was defined as the daily consumption of sugary drinks and/or consumption of fruits and/or vegetables less than several times daily and/or consumption of fast food and/or instant meals more than once a week.

**Research design and methods - Data handling**

**Calculation incidence rate**

The incidence rate was calculated using the following formula:

Incidence = [(new cases) / (population at risk x Follow-up time)]*1000

Of note: The incidence rate is expressed per 1000 years person follow-up time to simplify comparison.

The population at risk for developing the condition during the observation time (i.e., time between visit 1 and visit 2) were identified based on their glycaemic status at visit 1. This resulted in several groups: A = participants with NBG at visit 1 at risk for developing prediabetes, B = participants with NBG at visit 1 (at risk for developing any dysglycaemia, i.e., prediabetes or diabetes), and C = participants with NBG or prediabetes at visit 1 (at risk for developing diabetes).

The follow-up time of individual at risk (i.e., person-years at risk) was calculated. In case the individual at risk did not develop (A) prediabetes, (B) any dysglycaemia (i.e., prediabetes or diabetes), or (C) diabetes between visit 1 and 2, the full time between visits was calculated and used. If the individual at risk did develop (A) prediabetes, (B) any dysglycaemia (i.e., prediabetes or diabetes), or (C) diabetes between visit 1 and 2, half of the time in between visits was calculated and used.

The follow-up time * population at risk was calculated by summing the follow-up time of each individual at risk.

**References:**

Lampert T, Kroll LE, Müters S et al. (2013). Messung des sozioökonomischen Status in der Studie “Gesundheit in Deutschland aktuell“ (GEDA). Bundesgesundheitsbl 2013, 56, p. 131-143. DOI 10.1007/s00103-012-1583-3

The Federal Statistical Office of Austria ("Statistics Austria"). Household income. Available under: <https://www.statistik.at/en/statistics/population-and-society/income-and-living-conditions/household-income>. Accessed: 22th of October, 2024.
