# Supplementary figures and images for: Incidence of Prediabetes and Diabetes in a European Longitudinal General Population Cohort and Its Associated Factors—Results From the Austrian LEAD Study
Source: J Diabetes Res. 2025 Apr 22;2025:5540276. doi: 10.1155/jdr/5540276 (PMC12041627; doi:10.1155/jdr/5540276)

**Supplemental material – Online supplement 3**

**
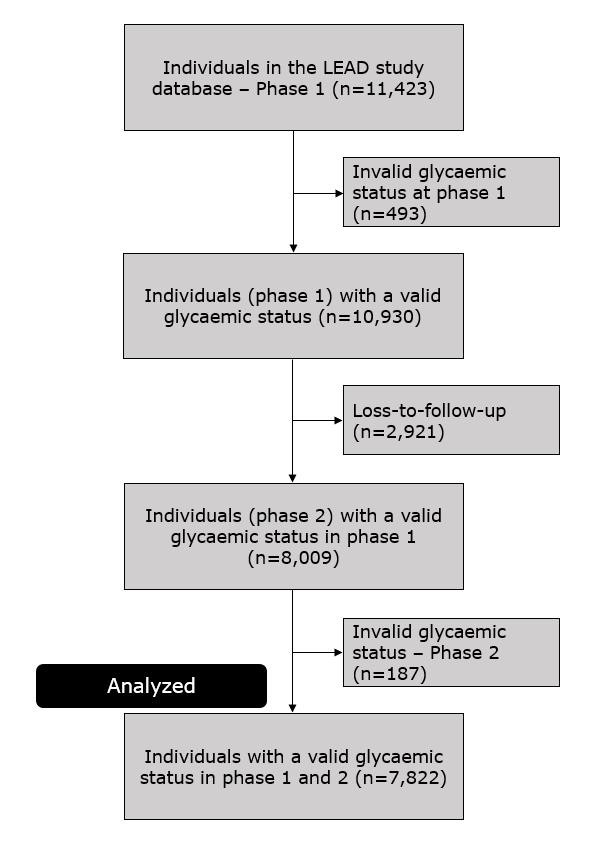
Online Figure 1.** Flowchart of patient selection.

Supplement: Supporting Information 3 — Figure S1: Flowchart of patient selection. [file 5540276.f3.docx]
