# Supplementary material for: Incidence of Prediabetes and Diabetes in a European Longitudinal General Population Cohort and Its Associated Factors—Results From the Austrian LEAD Study
Source: J Diabetes Res. 2025 Apr 22;2025:5540276. doi: 10.1155/jdr/5540276 (PMC12041627; doi:10.1155/jdr/5540276)
Supplement: Supporting Information 4 — Table S2: Characteristics of included and excluded individuals at Visit 1. [file 5540276.f4.docx]

**Supplemental material - Online supplement 4**

|  | n | Included Participants  (n=7822) | n | Excluded Participants  (n=3108) |
| --- | --- | --- | --- | --- |
| Female sex, n (%) | 7822 | 4016 (51.3) | 3108 | 1682 (54.1) |
| Age (years), mean (SD) | 7822 | 45.7 (18.5) | 3108 | 42.9 (21.2) |
| <18 years, n (%) |  | 751 (9.6) |  | 451 (14.5) |
| HbA1c in %, mean (SD) | 7807 | 5.3 (0.5) | 3095 | 5.3 (0.6) |
| Fasted blood glucose in mg/dl, mean (SD) | 7813 | 93.1 (15.5) | 3099 | 94.0 (19.5) |
| BMI in kg/m^2^, mean (SD) | 7071 | 25.9 (4.7) | 2657 | 26.2 (5.2) |
| Fitted BMI in kg/m^3^, mean (SD) | 751 | 12.5 (2.0) | 449 | 13.0 (2.4) |
| Obesity*, n (%) | 7812 | 1229 (15.7) | 3102 | 561 (18.1) |
| Waist circumference (cm), mean (SD) | 7811 | 91.7 (14.9) | 3100 | 90.6 (16.7) |
| Smoking (%) | 7817 |  | 3105 |  |
| Current smoker, n (%) |  | 1619 (20.7) |  | 751 (24.2) |
| Former smoker, n (%) |  | 2341 (29.9) |  | 783 (25.2) |
| Smoking history in packyears, mean (SD) | 7692 | 8.9 (16.9) | 3078 | 10.5 (21.1) |
| Regular alcohol consumer, n (%) | 7822 | 720 (9.2) | 3107 | 232 (7.5) |
| Low education level, n (%) | 7064 | 1925 (27.3) | 2649 | 960 (36.2) |
| Low household income, n (%) | 5765 | 664 (11.5) | 1983 | 396 (20.0) |
| Activity sum (min/day), mean (SD) | 7785 | 82.6 (90.0) | 3098 | 85.9 (92.9) |
| Unhealthy nutrition, n (%) | 7822 | 5902 (75.5) | 3107 | 2432 (78.3) |
| Rural residence, n (%) | 7822 | 1354 (17.4) | 3108 | 404 (13.0) |
| *Abbreviations: HbA1, glycated haemoglobin; BMI, body mass index. * Presence of obesity is based on a BMI ≥30 kg/m2 (adults) and as >2 standard deviations (SDs) above the mean BMI-for-age value mean (<18years).* | | | | |

**Online Table 2.** Characteristics of included and excluded individuals at visit 1.
