# Supplementary material for: Incidence of Prediabetes and Diabetes in a European Longitudinal General Population Cohort and Its Associated Factors—Results From the Austrian LEAD Study
Source: J Diabetes Res. 2025 Apr 22;2025:5540276. doi: 10.1155/jdr/5540276 (PMC12041627; doi:10.1155/jdr/5540276)
Supplement: Supporting Information 5 — Table S3: Incidence of prediabetes, any dysglycaemia, and diabetes per 1000 person-years in 10-year groups. [file 5540276.f5.docx]

**Supplemental material - Online supplement 5**

| **Age at visit 1** | **Glycaemic status** | **Cases** | **Person-years contributed by cases** | **Person-years at risk** | **Incidence [95%CI] in 1000 person-years** |
| --- | --- | --- | --- | --- | --- |
| 6-<10 | Prediabetes | 27 | 58.2 | 894.0 | 30.20 [18.81;41.59] |
|  | Any dysglycaemia | 29 | 62.4 | 889.8 | 32.59 [20.73;44.45] |
|  | Diabetes | 2 | 4.2 | 1001.0 | 2.00 [0.00;4.77] |
| 10-<20 | Prediabetes | 43 | 91.3 | 2639.8 | 16.29 [11.42;21.16] |
|  | Any dysglycaemia | 52 | 111.0 | 2620.1 | 19.85 [14.45;25.24] |
|  | Diabetes | 10 | 21.6 | 2823.3 | 3.54 [1.35;5.74] |
| 20-<30 | Prediabetes | 49 | 107.6 | 3652.3 | 13.42 [9.66;17.17] |
|  | Any dysglycaemia | 53 | 115.4 | 3644.5 | 14.54 [10.63;18.46] |
|  | Diabetes | 5 | 9.6 | 3997.3 | 1.25 [0.15;2.35] |
| 30-<40 | Prediabetes | 120 | 258.2 | 3908.6 | 30.70 [25.21;36.19] |
|  | Any dysglycaemia | 133 | 285.8 | 3881.0 | 34.27 [28.45;40.09] |
|  | Diabetes | 17 | 36.5 | 4503.3 | 3.78 [1.98;5.57] |
| 40-<50 | Prediabetes | 294 | 629.4 | 4517.1 | 65.09 [57.65;72.53] |
|  | Any dysglycaemia | 314 | 673.2 | 4473.3 | 70.19 [62.43;77.96] |
|  | Diabetes | 37 | 80.9 | 6086.3 | 6.08 [4.12;8.04] |
| 50-<60 | Prediabetes | 384 | 813.7 | 3492.9 | 109.94 [98.94;120.93] |
|  | Any dysglycaemia | 407 | 863.9 | 3442.6 | 118.22 [106.74;129.71] |
|  | Diabetes | 70 | 150.8 | 5873.1 | 11.92 [9.13;14.71] |
| 60-<70 | Prediabetes | 306 | 641.3 | 2194.9 | 139.41 [123.79;155.03] |
|  | Any dysglycaemia | 322 | 675.7 | 2160.4 | 149.05 [132.77;165.32] |
|  | Diabetes | 75 | 160.4 | 4612.9 | 16.26 [12.58;19.94] |
| 70+ | Prediabetes | 178 | 365.2 | 937.2 | 189.93 [162.02;217.83] |
|  | Any dysglycaemia | 188 | 388.3 | 914.2 | 205.65 [176.25;235.05] |
|  | Diabetes | 49 | 105.1 | 2480.8 | 19.75 [14.22;25.28] |

**Online Table 3.** Incidence of prediabetes, any dysglycaemia, and diabetes per 1000 person-years in 10 years groups.
