# Supplementary material for: Incidence of Prediabetes and Diabetes in a European Longitudinal General Population Cohort and Its Associated Factors—Results From the Austrian LEAD Study
Source: J Diabetes Res. 2025 Apr 22;2025:5540276. doi: 10.1155/jdr/5540276 (PMC12041627; doi:10.1155/jdr/5540276)
Supplement: Supporting Information 6 — Table S4: Incidence of prediabetes per 1000 person-years in 10-year groups stratified by sex. [file 5540276.f6.docx]

**Supplemental material - Online supplement 6**

**Online Table 4.** Incidence of prediabetes per 1000 person-years in 10 years groups stratified for sex.

| **Age at visit 1** | **Sex** | **Cases** | **Person-years contributed by cases** | **Person-years at risk** | **Incidence [95%CI] in 1000 person-years** |
| --- | --- | --- | --- | --- | --- |
| 6-<10 | Male | 17 | 36.9 | 491.4 | 34.60 [18.15;51.04] |
|  | Female | 10 | 21.3 | 402.6 | 24.84 [9.44;40.23] |
| 10-<20 | Male | 28 | 58.3 | 1238.0 | 22.62 [14.24;31.00] |
|  | Female | 15 | 32.9 | 1401.8 | 10.70 [5.29;16.12] |
| 20-<30 | Male | 30 | 65.2 | 1744.6 | 17.20 [11.04;23.35] |
|  | Female | 19 | 42.4 | 1907.7 | 9.96 [5.48;14.44] |
| 30-<40 | Male | 71 | 154.2 | 1905.4 | 37.26 [28.59;45.93] |
|  | Female | 49 | 104.1 | 2003.2 | 24.46 [17.61;31.31] |
| 40-<50 | Male | 146 | 312.6 | 1824.3 | 80.03 [67.05;93.01] |
|  | Female | 148 | 316.8 | 2692.8 | 54.96 [46.11;63.82] |
| 50-<60 | Male | 150 | 312.2 | 1442.5 | 103.99 [87.35;120.63] |
|  | Female | 234 | 501.5 | 2050.4 | 114.12 [99.50;128.75] |
| 60-<70 | Male | 126 | 264.4 | 839.5 | 150.08 [123.88;176.29] |
|  | Female | 180 | 376.9 | 1355.4 | 132.81 [113.40;152.21] |
| 70+ | Male | 84 | 171.8 | 474.4 | 177.08 [139.21;214.95] |
|  | Female | 94 | 193.4 | 462.9 | 203.09 [162.03;244.14] |
