# Supplementary material for: Incidence of Prediabetes and Diabetes in a European Longitudinal General Population Cohort and Its Associated Factors—Results From the Austrian LEAD Study
Source: J Diabetes Res. 2025 Apr 22;2025:5540276. doi: 10.1155/jdr/5540276 (PMC12041627; doi:10.1155/jdr/5540276)
Supplement: Supporting Information 7 — Table S5: Incidence of diabetes per 1000 person-years in 10-year groups stratified by sex. [file 5540276.f7.docx]

**Supplemental material - Online supplement 7**

| **Age at visit 1** | **Sex** | **Cases** | **Person-years contributed by cases** | **Person-years at risk** | **Incidence [95%CI] in 1000 person-years** |
| --- | --- | --- | --- | --- | --- |
| 6-<10 | Male | 0 | 0 | 555.9 | 0 [0.00;NA] |
|  | Female | 2 | 4.2 | 445.1 | 4.49 [0.00;10.72] |
| 10-<20 | Male | 5 | 10.9 | 1357.7 | 3.68 [0.45;6.91] |
|  | Female | 5 | 10.7 | 1465.6 | 3.41 [0.42;6.40] |
| 20-<30 | Male | 4 | 7.8 | 1965.6 | 2.03 [0.04;4.03] |
|  | Female | 1 | 1.8 | 2031.7 | 0.49 [0.00;1.46] |
| 30-<40 | Male | 13 | 26.4 | 2304.9 | 5.64 [2.57;8.71] |
|  | Female | 4 | 10.1 | 2198.3 | 1.82 [0.04;3.60] |
| 40-<50 | Male | 20 | 41.7 | 2772.5 | 7.21 [4.05;10.38] |
|  | Female | 17 | 39.2 | 3313.8 | 5.13 [2.69;7.57] |
| 50-<60 | Male | 41 | 85.9 | 2628.0 | 15.60 [10.83;20.38] |
|  | Female | 29 | 64.9 | 3245.1 | 8.94 [5.68;12.19] |
| 60-<70 | Male | 39 | 83.7 | 2033.7 | 19.18 [13.16;25.20] |
|  | Female | 36 | 76.8 | 2579.3 | 13.96 [9.40;18.52] |
| 70+ | Male | 27 | 57.9 | 1242.7 | 21.73 [13.53;29.92] |
|  | Female | 22 | 47.2 | 1238.1 | 17.77 [10.34;25.19] |

**Online Table 5.** Incidence of diabetes per 1000 person-years in 10 years groups stratified for sex.
