# Supplementary material for: Incidence of Prediabetes and Diabetes in a European Longitudinal General Population Cohort and Its Associated Factors—Results From the Austrian LEAD Study
Source: J Diabetes Res. 2025 Apr 22;2025:5540276. doi: 10.1155/jdr/5540276 (PMC12041627; doi:10.1155/jdr/5540276)
Supplement: Supporting Information 8 — Table S6: Incidence of any dysglycaemia (prediabetes or diabetes) per 1000 person-years in 10-year groups stratified for sex. [file 5540276.f8.docx]

**Supplemental material - Online supplement 8**

**Online Table 6.** Incidence of any dysglycaemia (prediabetes or diabetes) per 1000 person-years in 10 years groups stratified for sex.

| **Age at visit 1** | **Sex** | **Cases** | **Person-years contributed by cases** | **Person-years at risk** | **Incidence [95%CI] in 1000 person-years** |
| --- | --- | --- | --- | --- | --- |
| 6-<10 | Male | 17 | 36.9 | 491.4 | 34.60 [18.15;51.04] |
|  | Female | 12 | 25.5 | 398.4 | 30.11 [13.08;47.16] |
| 10-<20 | Male | 32 | 67.3 | 1229.0 | 26.04 [17.02;35.06] |
|  | Female | 20 | 43.7 | 1391.1 | 14.37 [8.08;20.68] |
| 20-<30 | Male | 34 | 73.0 | 1736.8 | 19.58 [13.00;26.16] |
|  | Female | 19 | 42.4 | 1907.7 | 9.96 [5.48;14.44] |
| 30-<40 | Male | 82 | 176.6 | 1883.0 | 43.55 [34.12;52.97] |
|  | Female | 51 | 109.2 | 1998.0 | 25.52 [18.52;32.53] |
| 40-<50 | Male | 155 | 331.1 | 1805.8 | 85.83 [72.32;99.35] |
|  | Female | 159 | 342.1 | 2667.5 | 59.61 [50.34;68.87] |
| 50-<60 | Male | 162 | 337.2 | 1417.5 | 114.28 [96.68;131.88] |
|  | Female | 245 | 526.7 | 2025.1 | 120.98 [105.83;136.13] |
| 60-<70 | Male | 134 | 281.1 | 822.8 | 162.85 [135.28;190.43] |
|  | Female | 188 | 394.7 | 1337.6 | 140.55 [120.46;160.64] |
| 70+ | Male | 92 | 190.4 | 455.8 | 201.86 [160.61;243.11] |
|  | Female | 96 | 197.9 | 458.4 | 209.42 [165.21;248.93] |
