# Supplementary material for: Incidence of Prediabetes and Diabetes in a European Longitudinal General Population Cohort and Its Associated Factors—Results From the Austrian LEAD Study
Source: J Diabetes Res. 2025 Apr 22;2025:5540276. doi: 10.1155/jdr/5540276 (PMC12041627; doi:10.1155/jdr/5540276)
Supplement: Supporting Information 9 — Table S7: Multivariate binary logistic regression analysis of the association between physical activity, serum markers, and anthropometrics in individuals developing a worse glycaemic status referent to individuals staying normoglycaemic in an adult population. Participants using statins in Visit 1 and/or 2 were excluded. [file 5540276.f9.docx]

**Supplemental Material – online supplement 9**

**Online Table 7.** Multivariate binary logistic regression analysis of the association between physical activity, serum markers and anthropometrics in individuals developing a worse glycaemic status referent to individuals staying normoglycaemic in an adult population. Participants using statins in visit 1 and/or 2 were excluded.

| **Variables** | **B estimate** | **SE** | **Z- statistic** | **OR [95%CI]** | **p-value** |
| --- | --- | --- | --- | --- | --- |
| (Intercept) | -4.185 | 0.207 | -20.171 | 0.015 [0.010;0.23] | **<.001** |
| Delta physical activity (min/day) | -0.000 | 0.000 | -0.810 | 1.000 [0.999;1.000] | .418 |
| Delta smoking (Pack years) | 0.000 | 0.005 | 0.093 | 1.000 [0.990;1.011] | .926 |
| Delta triglycerides (mg/dL) | 0.003 | 0.001 | 4.343 | 1.003 [1.002;1.005] | **<.001** |
| Delta HDL-C (mg/dL) | 0.009 | 0.004 | 2.080 | 1.009 [1.001;1.018] | **.038** |
| Delta hsCRP (mg/dL) | 0.016 | 0.011 | 1.447 | 1.016 [0.995;1.039] | .148 |
| Delta fibrinogen (g/L) | 0.000 | 0.082 | -0.005 | 1.000 [0.851;1.174] | .996 |
| Delta cholesterol (mg/dL) | -0.005 | 0.002 | -2.689 | 0.995 [0.992;0.999] | **.007** |
| Delta FMI (zscore) | -0.061 | 0.139 | -0.438 | 0.941 [0.717;1.235] | .661 |
| Delta LMI (zscore) | 0.092 | 0.150 | 0.614 | 1.096 [0.817;1.469] | .539 |
| Delta ALMI (zscore) | 0.032 | 0.111 | 0.292 | 1.033 [0.832;1.285] | .770 |
| Delta VAT mass (zscore) | 0.367 | 0.104 | 3.540 | 1.444 [1.177;1.768] | **<.001** |
| Sex (Female) | -0.242 | 0.089 | -2.724 | 0.785 [0.659;0.934] | **.006** |
| Age at visit 1 (Years) | 0.064 | 0.003 | 18.361 | 1.066 [1.059;1.074] | **<.001** |
| Household income at visit 1 (Low) | 0.135 | 0.144 | 0.935 | 1.144 [0.859;1.513] | .350 |
| Education level at visit 1 (Low) | 0.141 | 0.100 | 1.408 | 1.151 [0.945;1.400] | .159 |
| Place of residence at visit 1 (Rural) | -0.191 | 0.112 | -1.699 | 0.826 [0.661;1.028] | .089 |
| Nutrition at visit 1 (Unhealthy) | 0.220 | 0.100 | 2.193 | 1.246 [1.025;1.518] | **.028** |
| Delta is calculated by subtracting visit 1 from visit 2 (i.e., visit 2 minus visit 1). P-values in bold are significant (i.e., p-value <0.05). The model was adjusted for the following confounders: sex, age at Visit 1, household income at Visit 1, Education level at Visit 1, Place of residence at Visit 1, and Nutrition at Visit 1.  Abbreviations: ALMI, Appendicular Lean Mass Index; FMI, Fat Mass Index; HDL-C, high-density lipoprotein cholesterol; hsCRP, high-sensitivity C-reactive protein; LMI, Lean Mass Index; OR, Odds Ratio; VAT, Visceral Adipose Tissue; 95%CI, 95% confidence interval. | | | | | |
